# Retraction Note to: KLF5 regulated lncRNA *RP1* promotes the growth and metastasis of breast cancer via repressing p27kip1 translation

**DOI:** 10.1038/s41419-025-07630-z

**Published:** 2025-04-08

**Authors:** Xiaoting Jia, Lejuan Shi, Xiaorong Wang, Liyun Luo, Li Ling, Jiang Yin, Ying Song, Zhijie Zhang, Ni Qiu, Hao Liu, Min Deng, Zhimin He, Hongsheng Li, Guopei Zheng

**Affiliations:** Affiliated Cancer Hospital & Institute of Guangzhou Medical University, Key Laboratory of Protein Modification and Degradation, The State Key Laboratory of Respiratory, Guangzhou Key Laboratory of “Translational Medicine on Malignant Tumor Treatment”, Hengzhigang Road 78#, Guangzhou, 510095 Guangdong China

Retraction Note to: *Cell Death & Disease* 10.1038/s41419-019-1566-5, published online 9 May 2019

The Editors-in-Chief have retracted this article because of concerns regarding the figures presented in this work. These concerns call into question the article's overall scientific soundness. An investigation conducted after its publication discovered the following issues:

- two flow cytometry plots in Figure 2E, labelled *MDA-MB-231, sh-1#* and *MDA-MB-231, sh-2#*, appear to feature recurring patterns;
- two gel strips in Figure 3C, labelled *p27kip1, MDA-MB-231* and *p27kip1, BT549*, appear to overlap with each other, when re-scaled and rotated;
- a portion of Panel *MCF-7/RP1*, *Vec* in Figure 4C appears to overlap with a portion of Panel *MCF-7/sh-p27kip1, sh-ZEB1* in Figure 5F;
- a portion of Panel *MCF-7/sh-p27kip1, Vec* in Figure 5F appears to overlap, when rotated, with a portion of Panel *MCF-7/sh-p27kip1, ZEB1* in the same figure.

The Editors-in-Chief therefore no longer have confidence in the integrity of the research presented in this article.

The authors have not stated whether they agree or disagree with this retraction.

